# Phylogenomic analysis of glycogen branching and debranching enzymatic duo

**DOI:** 10.1186/s12862-014-0183-2

**Published:** 2014-08-23

**Authors:** Christian M Zmasek, Adam Godzik

**Affiliations:** 1 Bioinformatics and Systems Biology Program, Sanford-Burnham Medical Research Institute, 10901 N. Torrey Pines Road, La Jolla 92037, CA, USA

**Keywords:** Glycogen, Starch, Branching, Debranching, Glycogen storage disease, AGL, GBE1, GlgB, GlgX, TreX

## Abstract

**Background:**

Branched polymers of glucose are universally used for energy storage in cells, taking the form of glycogen in animals, fungi, Bacteria, and Archaea, and of amylopectin in plants. Some enzymes involved in glycogen and amylopectin metabolism are similarly conserved in all forms of life, but some, interestingly, are not. In this paper we focus on the phylogeny of glycogen branching and debranching enzymes, respectively involved in introducing and removing of the α(1–6) bonds in glucose polymers, bonds that provide the unique branching structure to glucose polymers.

**Results:**

We performed a large-scale phylogenomic analysis of branching and debranching enzymes in over 400 completely sequenced genomes, including more than 200 from eukaryotes. We show that branching and debranching enzymes can be found in all kingdoms of life, including all major groups of eukaryotes, and thus were likely to have been present in the last universal common ancestor (LUCA) but have been lost in seemingly random fashion in numerous single-celled eukaryotes. We also show how animal branching and debranching enzymes evolved from their LUCA ancestors by acquiring additional domains. Furthermore, we show that enzymes commonly perceived as orthologous, such as human branching enzyme GBE1 and *E. coli* branching enzyme GlgB, are in fact related by a gene duplication and consequently paralogous.

**Conclusions:**

Despite being usually associated with animal liver glycogen and plant starch, energy storage in the form of branched glucose polymers is clearly an ancient process and has probably been present in the last universal common ancestor of all present life. The evolution of the enzymes enabling this form of energy storage is more complex than previously thought and illustrates the need for explicit phylogenomic analysis in the study of even seemingly “simple” metabolic enzymes. Patterns of conservation in the evolution of the glycogen/starch branching and debranching enzymes hint at some as yet unknown mechanisms, as mutations disrupting these patterns lead to a variety of genetic diseases in humans and other mammals.

## 1 Background

In animals, glucose is stored as glycogen, whereas plants store glucose as starch. Starch is a mixture of α-amylose, a linear polysaccharide made of α(1–4) linked glucose molecules and amylopectin, a branched polysaccharide that varies from α-amylose by the presence of α(1–6) linked branches every 24 to 30 residues. Glycogen differs from amylopectin in that its α(1–6) branches occur more frequently, typically every 8 to 14 residues [1]. In animals, glycogen forms 100 to 400 Å diameter cytoplasmic granules, which in mammals are especially noticeable in cells that have the greatest need of glycogen—liver and muscle cells, but it is also produced in other types of cells, including neurons where it can have deleterious effects [2]. The branching is important for fast response to metabolic needs, because synthesis and degradation of the glycogen polymer can only occur from the non-reducing ends of the α-1,4 chains; therefore, highly branched glycogen has a higher number of “ends” per volume. Additionally, branching increases the water solubility of glycogen [3]–[6]. While the glycogen role in mammals is best known, it has also been shown to be used as a metabolic reserve in yeast and various bacteria [7].

**Figure 1:**
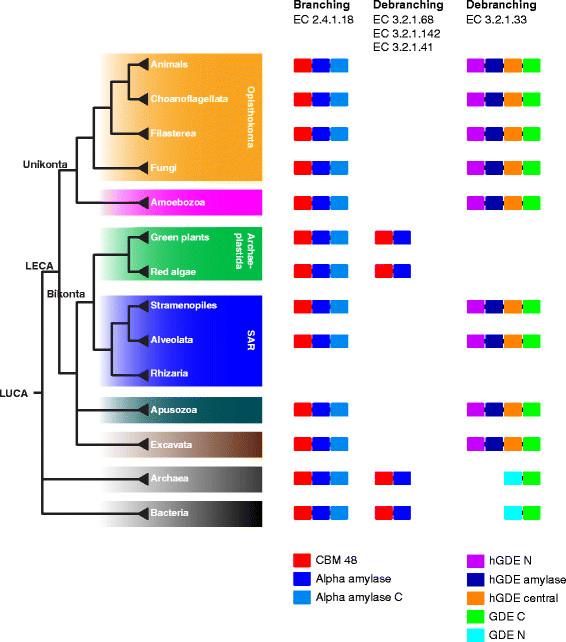
**Overview of the phylogenomic distribution of glycogen branching and debranching enzymes and their architectures.** The evolutionary tree is based on [10]–[12] (the placements of Apusozoa and Rhizaria are under debate).

Glycogen synthesis and breakdown involves a number of enzymes, such as glycogen synthase (EC 2.4.1.11), which adds glucose to the growing glycogen chain, and glycogen phosphorylase (EC 2.4.1.1), which cleaves linear α(1–4) linked glycogen chains to produce monomers of glucose-1-phosphate. The activity of glycogen phosphorylase, however, comes to a halt when it approaches an α(1–6) linked branch point four units away. In this situation, the action of a debranching enzyme, which removes α(1–6) linkages, becomes necessary for continued glycogen breakdown [6],[8]. Such debranching enzymes—together with their enzymatic opposites, branching enzymes that introduce α(1–6) linkages—are the focus of this work. Humans, and other mammals, possess one branching enzyme and one debranching enzyme, occurring in various isoforms [9] (see Figure 1 for an overview).

**Figure 2:**
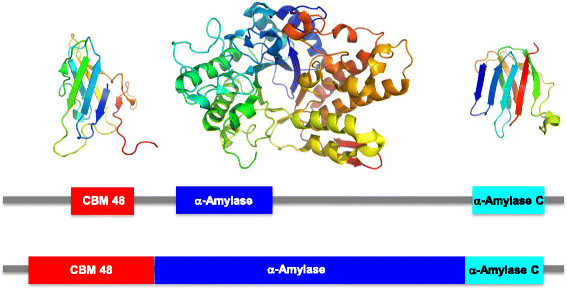
**Domain and three-dimensional structure of the human glycogen branching enzyme GBE1.** The three-dimensional structure for PDB entry 4bzy is shown in the top panel. Domain boundaries are shown according to Pfam (middle panel) and according to the 3D structure (lower panel). CBM_48 (boundaries are 75-162 according to Pfam, and 22-182 according to the 3D structure) is shown in red, Alpha_amylase (Pfam: 218-338, 3D: 183-599) in blue, and Alpha_amylase_C (Pfam: 603-698, 3D: 599-698) in cyan.

The human glycogen branching enzyme (EC 2.4.1.18), also referred to as Amylo-(1,4–1,6)-transglycosylase, is encoded by the gene *GBE1*. This enzyme is involved in glycogen synthesis by transferring α(1–4) linked glucosyl blocks from the outer end of a growing glycogen chain to an α(1–6) position on the same or on an adjacent chain [13]. Also, this enzyme (together with other glycogen and starch branching enzymes) has been characterized in the CAZy database [14] as a member of the Glycoside Hydrolase Family 57 [15],[16]. The human glycogen branching enzyme is a large, multidomain enzyme composed of three domains. The N-terminal domain of this enzyme is classified in Pfam [17] and CAZy as Carbohydrate-Binding Module 48 (also called Isoamylase N-terminal domain; and abbreviated as CBM_48) [18]. The central domain is a TIM barrel glycosyl hydrolase superfamily member (Pfam: Alpha-amylase) [19]; and localized at the C-terminus is an all-beta domain (Pfam: Alpha-amylase_C). The N- and C-terminal domains, CBM_48 and Alpha-amylase_C, respectively, are distantly homologous and structurally similar. Both are classified as members of the Glycosyl hydrolase domain (GHD) superfamily (Pfam clan CL0369), which contains substrate binding domains of many carbohydrate hydrolases. Branching enzymes with this domain architecture are well conserved throughout all kingdoms of life, with homologs possessing all three domains having been found in plants (as starch branching enzymes) [20], yeast [21], and various Bacteria, including *E. coli* (gene name *glgB*) [22]. The three dimensional structures of human (PDB identifier: 4BZY) as well as a variety of bacterial (for example, *E. coli*: 1M7X [23], *Mycobacterium tuberculosis*: 3K1D [24]) and archaeal (*Thermococcus kodakarensis*: 3N8T, 3 N92, 3 N98 [15]) glycogen branching enzymes, together with plant starch branching enzymes (rice *Oryza sativa*: 3AMK [25]), were determined experimentally, allowing for precise domain boundary definitions and detailed comparisons. For the human glycogen branching enzyme, these data are shown in Figure 2 which depicts the three-dimensional structure of the GBE1 gene product, in concert with domain boundaries as defined by Pfam HMMs and by the three-dimensional structure itself.

The human glycogen debranching enzyme, is encoded by the gene *GDE* (also called *AGL*). This enzyme, similar to its homologs from some other species (such as other mammals, yeast, and TreX from *Sulfolobus acidocaldarium*[26],[27]), has two biochemical functions—that of amylo-alpha-1,6-glucosidase (EC 3.2.1.33) and of 4-alpha-glucanotransferase (EC 2.4.1.25) [28]–[30]. 4-alpha-glucanotransferase transfers a segment of three glucose units from α(1–6) branched four-unit chains (the result of glycogen phosphorylase activity) to an adjacent branch of the glycogen chain. Amylo-alpha-1,6-glucosidase then cleaves the α(1–6) linkage to release the remaining glucose [8]. In other species, such as plants and *E. coli* (GlgX [31]), the glucosidase and glucanotransferase activities are carried out by two distinct enzymes (despite the high structural similarity between the *E. coli* glucosidase GlgX and *Sulfolobus acidocaldarium* TreX [32]), in which case only the glucosidase is referred to as a glycogen debranching enzyme [28]–[30].

**Figure 3:**
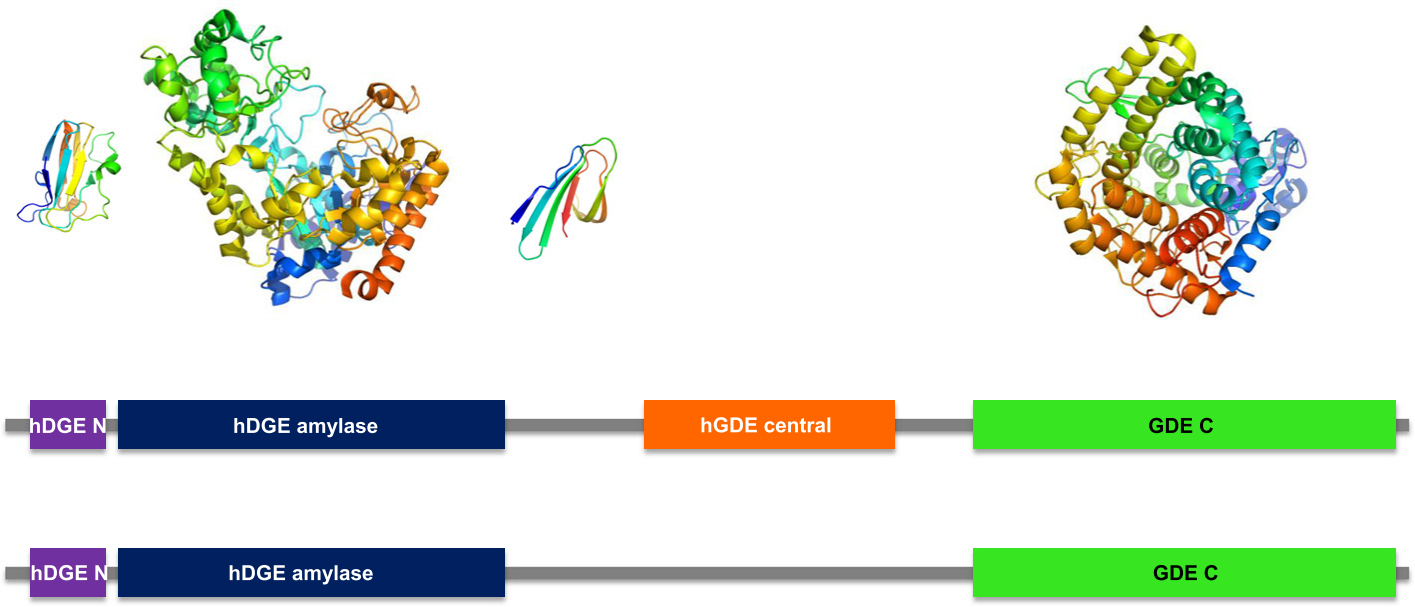
**Domain and predicted three-dimensional structure of human the glycogen debranching enzyme (GDE).** No experimental structure for human GDE or any close homologs is available as of this writing; however, three out of four predicted domains can be reliably predicted. Note that no reliable prediction can be made for the hDGE_central domain (orange) and that it is likely that this region corresponds to several small domains. One of the possible domains in this region is shown. Domain boundaries according to Pfam (middle panel) and according to the predicted 3D structure (lower panel) are shown as well. hDGE_N (30-117) is shown in purple, hDGE_amylase (120-550) in dark blue, hGDE_central (Pfam: 697-975) in orange, and GDE_C (1044-1527) in green.

The human glycogen debranching enzyme is almost twice as large as the GBE1 enzyme and is composed of at least four domains (using Pfam classification): hGDE_N—hGDE_amylase—hGDE_central—GDE_C. The hGDE_amylase domain and the central domain of the branching enzyme, Alpha-amylase, are distantly related, as both are members of the TIM barrel fold containing the glycosyl hydrolase superfamily (Pfam clan CL0058) [33],[34]. On the other hand, the N_terminal DGE_C domain is predicted to have an alpha/alpha toroidal structure, consisting of several alpha hairpins arranged in a closed circular array, similar to bacterial glucoamylases. As of this writing, there are no experimentally determined three-dimensional structures of human debranching enzymes or any of its close homologs, albeit, as shown in Figure 3, reliable predictions can be made for all its domains, except the hGDE_central domain.

In contrast to the universally conserved branching enzyme, some bacterial—for instance *E. coli* (GlgX) [35], and plant debranching enzymes [36]—are not homologous to the human debranching enzyme. In fact, they are related to the branching enzymes containing an N-terminal CBM_48 domain followed by Alpha-amylase domains. On the other hand, many Bacteria and Archaea do not have *E. coli–*type debranching enzymes; instead, they have a homolog of the human debranching enzyme, consisting of the prokaryote-specific C-terminal GDE domain preceded by a GDE_N domain. No direct experimental evidence for the function of the bacterial proteins with the GDE domain currently exists; however, because of their distant homology to eukaryotic debranching enzymes and their genomic distribution, where they are often found in species that lack the *E. coli*–type of a debranching enzyme, it is often assumed that they indeed function as debranching enzymes. As mentioned above, another difference between human and plant/*E. coli* debranching enzymes is that human glycogen debranching enzyme possesses a second enzymatic activity, that of a 4-alpha-glucanotransferase [28]–[30].

### 1.1 Glycogen storage diseases

Enzymes involved in glycogen metabolism and its regulation are of great medical interest because mutations in these enzymes have been shown to lead to a wide variety of genetic diseases, collectively called glycogen storage diseases (GSDs). At least ten different types (identified with numerical sub-type designations) of GSDs have been described, with the phenotypes depending on the enzyme affected and the specific positions of the mutations within a given enzyme. Some of the mutations result in the abnormal accumulation of glycogen and/or abnormal glycogen structure (or both). For example, Cori’s disease (Type III GSD), a rare autosomal recessive genetic disorder, is caused by mutations resulting in deficiencies in the glycogen debranching enzyme, preventing depolymerization of glycogen at the α-1,6 branching points and result in the accumulation, in the liver and muscle, of abnormal glycogen with very short outer chains that cannot be broken down further. The symptoms, such as enlarged liver and hypoglycemia, are similar, but tend to be less severe, than those of Type I GSD. Interestingly, the liver symptoms usually disappear after puberty [37],[38]. One of the most severe glycogen storage diseases is Anderson’s disease (Type IV GSD) [39]–[41], a very rare autosomal recessive genetic disorder caused by a defective glycogen branching enzyme (EC 2.4.1.18), leading to the formation and accumulation of abnormal glycogen with long, unbranched chains. GSDIV is also called amylopectinosis, since the glycogen in the affected cells resembles plant amylopectin. The abnormal, elongated glycogen particles lead to cell degeneration and eventually death. The exact mechanism of the cell death in GSDIV is still unknown. The phenotype of this disease is variable, involving the liver, skeletal muscle, heart, and central nervous system, alone or in combinations. The typical GSD Type IV disease presents in the first 18 months of life with an enlarged liver and cirrhosis, leading to liver failure and death by 5 years of age [42]. Another manifestation of the same disease, in the late-onset variant, is as a neurogenerative disease called adult polyglucosan body disease (APBD) [43]. GSDIV is also found in horses and cats [13],[44]. Table 1 lists some of the point mutations causing GSDIII, GSDIV and APBD.

**Table 1:** Human disease mutations in GSD3 and GSD4 and their counterparts in other species

		**Branching enzyme (EC 2.4.1.18)**									**Debranching enzyme (EC 3.2.1.33)**			
Human disease				GSD4			GSD4/APBD		GSD4				GSD3	
Human disease mutation position				224	257	329	515	524	545	628			1147	1448
Human disease mutation				L-P	F-L	Y-S	R-C/H	R-Q	H-R	H-R			R-G	G-R
		Protein Acc.	Gene	Alpha-amylase			Intra-domain			AC	Protein Acc.	Gene	GDE_C	
Animals	*H. sapiens*	Q04446	*GBE1*	L	F	Y	R	R	H	H	P35573	*AGL*	R	G
	*M. musculus*	Q9D6Y9	*Gbe1*	L	F	Y	R	R	H	H	F8VPN4		R	G
	*D. rerio*	XP_687620		L	F	Y	R	R	H	H	NP_001166124		R	G
	*C. elegans*	Q22137		L	F	Y	R	R	H	H	O62334	*agl-1*	R	G
	*D. melanogaster*	A1Z992	*AGBE*	V	Y	Y	R	R	H	H	E1JGQ5		R	G
	*A. queenslandica*	I1FQH3		L	H	Y	R	R	H	H	I1FDE0		R	G
Choanoflagellida	*M. brevicollis*	A9URY2		L	F	Y	R	R	H	H	A9V544		R	G
Ichthyosporea	*C. owczarzaki*	E9C2E3		L	F	Y	R	R	H	H	E9CDY8		R	G
Filasterea	*S. arctica*	09810 T0		I	F	Y	R	R	H	H	05895 T0		R	G
Fungi	*S. cerevisiae*	P32775	*GLC3*	L	F	Y	R	R	H	H	Q06625	*GDB1*	R	G
Amoebozoa	*D. discoideum*	Q555Q9	*glgB*	L	F	Y	R	R	H	H	Q54K94	*agl*	R	G
Land Plants	*A. thaliana*	O23647	*SBE2.1*	L	F	Y	R	R	H	H				
		Q9LZS3	*SBE2.2*	L	F	Y	R	R	H	H				
	*O. sativa*	Q01401	*SBE1*	L	F	Y	R	H	H	H				
		Q6H6P8		L	F	Y	R	R	H	H				
Green Algae	*V. carteri*	D8TIE8	*glgb6*	L	F	Y	R	R	H	H				
		D8U9K6	*glgb7*	L	F	Y	R	R	H	S				
Red Algae	*C. merolae*	CMH144C		L	F	Y	R	R	H	H				
Alveolata	*P. tetraurelia*	A0DXF8		L	F	Y	R	R	H	H	A0BWA0		R	G
	*T. thermophila*	Q23TC5		L	Y	F	K	R	S	N	I7M1C6		R	G
Apusozoa	*T. trahens*	09093T0		L	F	Y	R	R	H	H	02577T0		R	G
Bacteria	*B. theta*	Q8A9P4		L	F	Y	R	R	H	N	Q89ZS3		E	G
	*E. coli*	P07762	*glgB*	V	Y	Y	P	N	Q	T				
	*N. punctiforme*	B2J3N1	*glgB*	I	Y	Y	P	N	Q	T				

Human disease (missense) mutations responsible for the glycogen storage diseases GSD4, adult polyglucosan body disease (APBD), and GSD3, and their homologous residues in a variety of species are shown. The domains affected by the mutations are indicated in the 5^th^ row (AC stand for Alpha-amylase_C). *B. theta* stands for *Bacteroides thetaiotaomicron*. Human mutation data were obtained from the UniProtKB and Online Mendelian Inheritance in Man databases (OMIM).

Although phylogenetic studies have been performed on subsets of branching and debranching enzymes from a limited range of species (e.g., [20],[45],[46]), and experimental work (biochemistry, gene expression, protein structures, and biotechnological applications [47]) has been done on individual branching and debranching enzymes, especially those from plants, Bacteria, and Archaea, not much is known about the deep evolutionary histories of these enzyme families. The objective of the analysis presented here is to elucidate the evolution of glycogen and starch branching and debranching enzymes from a wide range of species, covering Bacteria, Archaea, and all major groups of eukaryotes. In particular, due to their involvement in glycogen storage diseases, we are interested in the evolutionary relationships of the human glycogen branching and debranching enzymes to their well-studied bacterial counterparts GlgB and GlgX/TreX. Due to the availability of more than 200 completely sequenced eukaryotic genomes, we were able to perform a large scale, protein domain-centric, comparative genomics analysis to assess the linage specific distributions, domain compositions and patterns of sequence conservation of these two important enzymes.

## 2 Results and discussion

We extracted protein sequences of glycogen branching and debranching enzyme homologs with the characteristic combinations of CBM48 and GDE_C Pfam domains (using a per domain cutoff E-value of 10^–3^) from 276 completely sequenced eukaryotic genomes, covering most major eukaryotic groups, as well as from select archaeal and bacterial genomes (listed in Additional file 1). Proteins with these domains were then analyzed for their overall domain architectures and for their phylogenetic relationships (listed in Additional files 2 and 3).

### 2.1 CBM_48—Alpha–amylase containing branching and debranching enzymes

**Figure 4:**
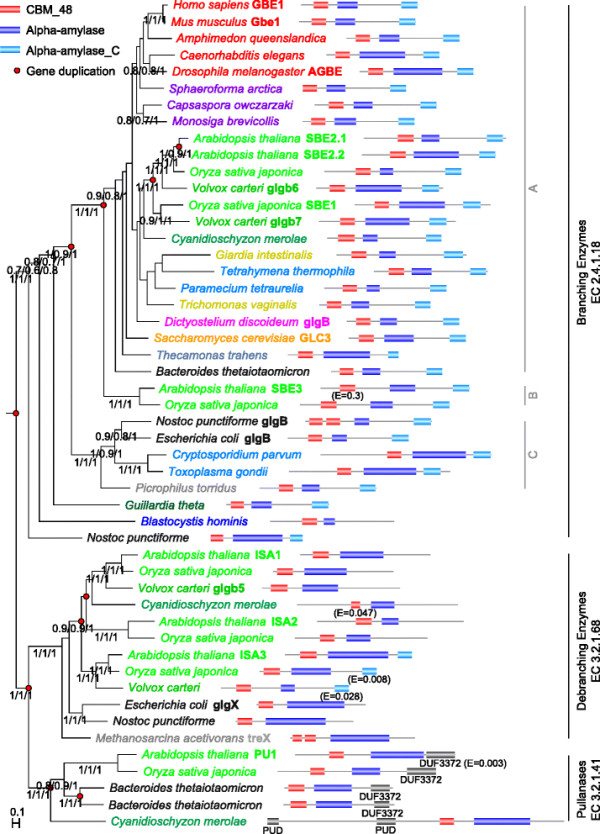
**Bayesian phylogeny of CBM_48—Alpha-amylase containing branching and debranching enzymes.** Only select protein names are shown (such as human GBE1 and *E. coli* GlgB and GlgX). The CBM_48 domain is shown in red, Alpha-amylase in blue, Alpha-amylase_C in light blue, and DUF3372 and PUD in gray. The E-value cutoff used for domains was 10^-3^ (exceptions are indicated in parentheses). For this figure, only representative species were analyzed (see Additional file 4); some taxonomy-dependent colors are: red—animals, bright green—green plants, light blue—Alveolata, light gray—Archaea, dark gray—Bacteria. The tree shown was inferred by MrBayes [49] based on a MAFFT [50] multiple sequence alignment. The support values shown are: minimal-evolution based bootstrap values normalized to 1.0 (ML distances calculated by TREE-PUZZLE [51], tree inference by FastME [52]) /ML based probabilities inferred by PhyML [53] /posterior probabilities calculated by MrBayes. Support values are only shown for branches for which *all* three values are at least 0.5. Branch length distances are proportional to expected changes per site. High-confidence gene duplications are shown as red circles [54].

Phylogenetic analysis of enzymes with a CBM_48—Alpha-amylase architecture (see Figure 4) shows that these enzymes can be divided into two well separated groups (100% support based on Bayesian, ML, and distance based methods): branching enzymes with a CBM_48—Alpha-amylase—Alpha-amylase_C architecture and debranching enzymes with mostly a CBM_48—Alpha-amylase architecture with either a very divergent form of the Alpha-amylase_C domain, or, in some cases, containing additional domains at the N- (such as the Bacterial pullanase-associated domain, PUD [48]) and C-termini (such as DUF 3372).

Proteins from the first group, with the well-defined CBM_48—Alpha-amylase—Alpha-amylase_C architecture are present in species from all kingdoms of life and perform branching functions in glycogen and starch biosynthesis pathways (EC 2.4.1.18). This group includes human GBE1, yeast GLC3, *Dictyostelium* GlgB, *Arabidopsis* SBEs, and *E. coli* GlgB. Most organisms have just one representative of this group, with the exception of land plants and green algae (Viridiplantae, “green plants”) and the photosynthetic cyanobacteria that tend to contain multiple paralogs from this group. For instance, in land plants three sub-groups of starch branching enzymes exist, usually named SBE1, SBE2, and SBE3. However, not all plants possess one member of each subgroup; for example, *Arabidopsis* underwent a recent duplication of SBE2, resulting in SBE2-1 and SBE2-2, and also has one SBE3 member, but lacks a representative of SBE1. Careful phylogenetic analysis shows that this group, due to at least two ancient gene duplications, one of which occurred pre-LUCA (last universal common ancestor) and one pre-LECA (last eukaryotic common ancestor), followed by lineage specific gene losses, has to be divided into a minimum of three sub-groups of orthologous proteins (labeled A, B, and C in Figure 4). While the existence of a separate, plant-specific subgroup containing SBE3 has been reported previously [20],[46], our results show that the well-studied *E. coli* branching enzyme GlgB, together with GlgB from the cyanobacterium *Nostoc punctiforme*, are clearly not orthologous to human GBE1, yeast GLC3, *Dictyostelium* GlgB, and plant SBE1, SBE 2, and SBE 3, but instead represent a branch that emerged by an ancient duplication and was lost in most eukaryotes. On the other hand, other bacterial branching enzymes, such as the one of *Bacteroides thetaiotaomicron* (UniProt: Q8A9P4), are indeed orthologous to human GBE1. We employed the RIO approach (Resampled Inference of Orthologs) [55],[56] on MrBayes [49] output gene trees to confirm these findings. In short, this approach allows to calculate the probability of orthology relationships by integrating orthology assignments over a distribution of gene trees (produced by MrBayes, in this case) [57]. According to this, the posterior probability of *E. coli* GlgB being orthologous to human GBE1 is 0.0, whereas the posterior probability of *Bacteroides thetaiotaomicron* Q8A9P4 of being orthologous to human GBE1 is 1.0. These results are further supported by analysis of conserved residues, as described below (see Table 1). Finally, it is likely that this group was affected by even more basal gene duplications, but due to relatively poor phylogenetic resolution at the base of this sub-tree, this remains speculative at this moment.

Enzymes from the second main group, with CBM_48—Alpha-amylase architectures, are only found in land plants and green algae, red algae (Rhodophyta), and some bacterial and archaeal species. Phylogenetic analysis further subdivides this group into two sub-groups of orthologous proteins, correlating with their annotated functions (100% support based on Bayesian, ML, and distance based methods). Enzymes in one sub-group perform debranching functions in glycogen and starch catabolic pathways (EC 3.2.1.68). Similar to branching enzymes, these enzymes underwent expansion in land plants. For example, *Arabidopsis* and *Oryza sativa japonica* (rice) contain three paralogs—Isoamylase 1, 2, and 3. The *E. coli* glycogen debranching enzyme GlgX is a member of this group as well. Pullanases (EC 3.2.1.41) form the second sub-group. These enzymes are found in land plants and green algae, red algae, and Bacteria (we were unable to detect any likely archaeal orthologs in our set of complete genomes) and have additional domains (Bacterial pullanase-associated domain, PUD [48], or DUF3372). The significantly different lengths of the Alpha_amylase domain of different species depicted in Figure 4 are likely artifacts of the Pfam HMM used to identify them. The three dimensional structure of the human glycogen branching enzyme shows that Alpha_amylase occupies most of the space between CBM_48 and Alpha_amylase_C (Figure 2). This is likely to be the case in all species.

### 2.2 GDE_C domain containing debranching enzymes

**Figure 5:**
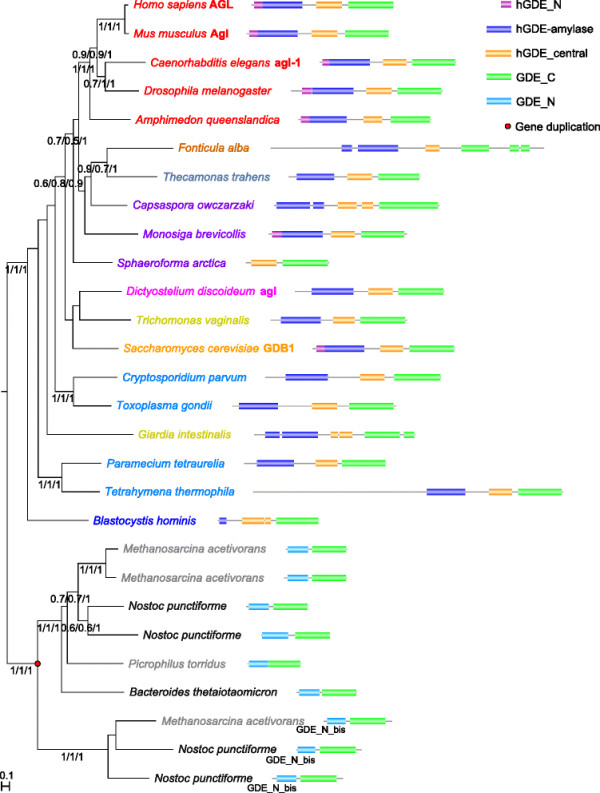
**Bayesian phylogeny of GDE_C domain containing (putative) debranching enzymes (EC 3.2.1.33).** Only select protein names are shown (such as human AGL and yeast GDB1). The GDE_C domain is shown in bright green, GDE_N in light blue, hGDE-amylase in blue, hGDE_central in orange, and hGDE_N in purple. The E-value cutoff used for domains was 10^-3^. For this figure, only representative species were analyzed (see Additional file 4); some taxonomy dependent colors are: red—animals, light blue—Alveolata, light gray—Archaea, dark gray—Bacteria. The tree shown was inferred by MrBayes [49] based on a MAFFT [50] multiple sequence alignment. The support values shown are: minimal evolution based bootstrap values normalized to 1.0 (ML distances calculated by TREE-PUZZLE [51], tree inference by FastME [52]) /ML based probabilities inferred by PhyML [53] /posterior probabilities calculated by MrBayes. Support values are only shown for branches for which *all* three values are at least 0.5. Branch length distances are proportional to expected changes per site. High-confidence gene duplication is shown as red circle [54].

Phylogenetic analysis of the debranching enzymes paints a very different picture from that of branching enzymes (see Figures 1 and 5). These proteins are found in animals and fungi and their relatives (members of the Unikonta) and in certain single-celled eukaryotes form the Bikonta group (such as *Paramecium tetraurelia*), as well as in Bacteria and Archaea, but are not present in land plants, green algae, and Rhodophyta (which are all members of the group Archaeplastida). They exhibit diverse domain architectures, especially between eukaryotes and Bacteria/Archaea. In eukaryotes, the Pfam domain architecture is generally hGDE_N—hGDE-amylase—hGDE_central—GDE_C, whereas bacterial and archaeal enzymes are much shorter and have a different domain architecture, with a GDE_N domain substituting for the three N-terminal domains of the eukaryotic enzymes, resulting in a GDE_N—GDE_C arrangement. As mentioned above, no direct experimental evidence for the function of these bacterial and archaeal proteins currently exists, although because of their distant homology to eukaryotic debranching enzymes and their genomic distribution, we speculate that they function as debranching enzymes. Furthermore, no three-dimensional structure of any protein from this group of bacterial and archaeal GDE_N—GDE_C enzymes is available as of this writing, and no reliable predictions can be made about possible relationships between hGDE_central and GDE_N domains and any other protein domains. A preliminary of analysis of the draft genome of *Cyanophora paradoxa*[58] indicates that this representative of the Glaucophyta (a small group of freshwater algae [59] which, together with Rhodophyta, are estimated to be the earliest branching members of Archaeplastida [60]) contains a putative debranching enzyme with a hGDE_central—GDE_C architecture (see Additional file 3) and thus is conceivable to have a pattern of branching/debranching enzymes dissimilar to that of other Archaeplastida. More genomic data from Glaucophyta will be needed to precisely determine where and when during Archaeplastida evolution the loss of the hGDE-amylase, hGDE_central, GDE_C, and hGDE_N domains occurred.

### 2.3 Distribution of branching and debranching enzymes in major groups of eukaryotes

**Figure 6:**
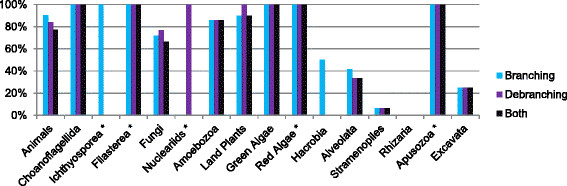
**Distribution of branching and debranching enzymes in major groups of eukaryotes.** Percentages of completely sequenced genomes with at least one branching enzyme, at least one debranching enzyme, and at least one of each (“Both”) are shown. Branching enzymes are defined by their CBM_48—Alpha-amylase domain architecture, debranching enzymes are of either CBM_48—Alpha-amylase or hGDE_N—hGDE-amylase-hGDE—hGDE-central—GDE_C architecture. Distinction between CBM_48—Alpha-amylase branchers and debranchers is based on phylogenetic analysis. A domain cutoff E-value of 10^-3^ was used. Groups marked with an asterisk are only represented by one fully sequenced genome (see Additional file 1).

We also investigated the distribution of branching and debranching enzymes over all major groups of eukaryotes with at least one completely sequenced genome (see Figure 6 for percentages, and Figure 1 for a simplified overview). The result is that branching and debranching enzymes can be found in all major groups of eukaryotes. The only *possible* exception to this is Rhizaria (a large group of mostly unicellular eukaryotes [61]), even though with only two completely sequenced genomes in this group, a conclusive answer is impossible at this point. Animals (and their closest relatives, the single-celled choanoflagelates), land plants, and green algae have the highest percentage of genomes with both branching and debranching enzymes (we suspect that the real number is close to 100%; missing enzymes in either category in some animal and plant genomes are mostly likely due to sequencing, assembly, and gene prediction errors and do not represent actual gene losses, as the “losses” appear randomly). For fungi and Amoebozoa, these percentages are lower but are still above 60% and 80%, respectively. On the other hand, the majority of the Alveolata, stramenopiles, and Excavata lack both enzymes but still contain some species with both enzymes (see Additional 4). For other groups, due to limited genomes sequenced, a reliable percentage cannot yet be calculated.

### 2.4 Human disease mutations in GSD3 and GSD4 and their counterparts

Finally (see Table 1), we investigated the amino-acid conservation in the positions mutated in the human glycogen storage diseases GSD3, GSD4, and APBD in orthologs of the human proteins (i.e. from sub-tree “A” for CBM_48—Alpha-amylase branching enzymes; see Figure 4) from a wide variety of species, as well as in the paralogous GlgB enzymes from *E. coli* and *Nostoc punctiforme*. Our results show that all disease mutations occur in highly conserved positions, even when compared to species as distantly related as plants and bacteria, stressing the importance of these positions/residues (strong conservation of the glycine at position 1448 in the human glycogen debranching enzyme, mutated in GSD3, has been noted previously [62]). On the other hand, in the case of CBM_48—Alpha-amylase branching enzymes, this conservation is not maintained in the paralogous enzymes from *E. coli* and *Nostoc punctiforme*. We have no explanation for unexpectedly low conversion in these positions in the putative enzyme from *Tetrahymena thermophila*, especially since our phylogenetic analysis reveals nothing unusual in its sequence.

## 3 Conclusions

Branching enzymes (EC 2.4.1.18) with a CBM 48—Alpha-amylase—Alpha-amylase C architecture are present in all the major group of eukaryotes, as well as in Archaea and Bacteria, and are therefore likely to have been present in the last universal common ancestor (LUCA). While they are found in the vast majority of all animal and plant genomes (including green algae) sequenced so far, and are fairly common in fungi, many individual species of single-celled eukaryotes lack identifiable homologs of these enzymes, likely due to gene loss.

For debranching enzymes (EC 3.2.1.68 and EC 3.2.1.142) with a CBM 48—Alpha-amylase architecture, the distribution is very different. On the eukaryotic side, these enzymes are limited to plants and green algae (for which they are found in the vast majority of all sequenced genomes). They are also fairly widespread in Bacteria and Archaea. In contrast, non-homologous debranching enzymes (EC 3.2.1.33) containing the GDE_C domain can be found in species from all kingdoms of life, *except* green plants and algae.

Comparing these two families allows us to conclude, that in plants, GDE_C-containing debranching enzymes have been replaced by CBM48-containing enzymes. In certain Bacteria (e.g. *Nostoc punctiforme*) both types of debranching enzymes exist in parallel.

The only major eukaryotic groups for which we are unable to make reliable conclusions are Rhizaria and Glaucophyta. Rhizaria is the only major group of eukaryotes with at least two fully sequenced genomes for which we were unable to detect any glycogen/starch branching or debranching enzymes (such enzymes could be found neither in the two completely sequenced species from Rhizaria, *Bigelowiella natans* and *Reticulomyxa filosa*, nor by searching for homologs in Rhizaria in UniProt and Genbank). Despite their importance in the study of plant evolution (due to their placement at the root of the Archaeplastida sub-tree), only one draft genome for Glaucophyta has been released as of this writing; preventing us from conclusively determining whether the pattern of branching/debranching enzymes in this group of alga is indeed not like that of plants and more like that of the rest of eukaryotes (as our preliminary results indicate).

As for human glycogen branching enzyme GBE1, the evolutionary history of this protein can be traced back to Bacteria, for a putative ortholog of human branching enzyme exists in several bacteria, for instance, in a dominant human gut symbiont—*Bacteroides thetaiotaomicron*. In contrast, our study shows that the well-known *E. coli* branching enzyme GlgB is *not* an ortholog of its human homolog, but a member of a separate branch that has been lost in many eukaryotes (including mammals). This obeservation, combined with the low conservation of residues mutated in human diseases in *E.coli* GlgB, has implications against its use as a model for studying human GSD4/APBD. On the other hand, the *Bacteroides thetaiotaomicron* branching enzyme is an attractive target for modeling human glycogen storage diseases in Bacteria.

Finally, these results show that not only regulatory proteins, such as those involved in apoptosis regulation [63], but also basic metabolic enzymes may have a complex evolutionary history, rich in ancient and recent gene duplications, combined with lineage specific gene losses and dynamic domain architectures, with frequent and surreptitious addition and loss of individual domains. Such a history can only be revealed by explicit phylogenetic and comparative domain architecture analysis.

## 4 Methods

### 4.1 Genomes

Protein predictions for organisms with a completely sequenced genome were obtained from the sources listed in Additional file 1, covering the following species: 93 animals, 2 choanoflagellates, *Capsaspora owczarzaki*, *Sphaeroforma arctica*, 78 fungi, *Fonticula alba*, 7 amoebozoans, 30 land plants, 10 green algae, *Cyanidioschyzon merolae*, *Cyanophora paradoxa* (draft), *Emiliania huxleyi*, *Guillardia theta*, 12 Alveolata, 16 Stramenopiles, *Bigelowiella natans*, *Reticulomyxa filose*, *Thecamonas trahens*, 8 Excavata, 49 Archaea, and 133 Bacteria. Hmmscan from HMMER 3.0 [64] together with HMMs for Carbohydrate-binding module 48 (Isoamylase N-terminal domain, CBM_40, PF02922) and Amylo-alpha-1,6-glucosidase (GDE_C, PF06202) from Pfam 27.0 [17] were used to extract putative branching and debranching enzyme sequences. We experimented with different per-domain E-value thresholds to ensure that the results presented here are robust and not simply artifacts of an arbitrarily chosen threshold. For the phylogenetic analyses we generally used a per-domain E-value threshold of 10^-3^ (unless noted otherwise).

### 4.2 Multiple sequence alignments

Multiple sequence alignments were calculated using MAFFT 7.017b (with “localpair” and “maxiterate 1000” options) and ProbCons 1.12 (default options) [65]. Prior to phylogenetic inference, multiple sequence alignment columns with more than 50% gaps were deleted; for comparison we also performed the analyses based on alignments for which we only deleted columns with more than 90% gaps.

### 4.3 Phylogenetic analyses

Distance-based minimal evolution trees were inferred by FastME 2.0 [52] (with balanced tree swapping and “GME” initial tree options) based on pairwise distances calculated by TREE-PUZZLE 5.2 [51] (using the WAG substitution model [66] as recommended by PROTTEST 1.4 [67], a uniform model of rate heterogeneity, estimation of amino acid frequencies from the dataset, and approximate parameter estimation using a Neighbor-joining tree). For maximum likelihood and Bayesian approaches we employed PhyML 2.4.4 [53] (using 100 bootstrapped data sets, the WAG substitution model, 4 substitution rate categories, estimated proportion of invariable sites, estimated Gamma distribution parameter, and an initial tree calculated by the BIONJ algorithm) and MrBayes 3.2.2 [49] (with 10^6^ generations, a sample frequency of 100, a mixture of amino-acid models with fixed rate matrices and equal rates, and 25% burn-in). For the calculations of typed support values from different sources, confadd 1.01 was used [56]. Tree and domain composition diagrams were drawn using Archaeopteryx [56]. All conclusions presented in this work are robust relative to the alignment methods, the alignment processing, the phylogeny reconstruction methods, and the parameters used. All sequence, alignment, and phylogeny files are available upon request.

### 4.4 Availability of supporting data

The data sets supporting the results of this article are available in the Dryad repository, doi:10.5061/dryad.34vq1, http://dx.doi.org/10.5061/dryad.34vq1[54], in phyloXML format [68].

## Abbreviations

APBD: Adult polyglucosan body disease

CAZy: Carbohydrate-active enzymes database

CBM: Carbohydrate-binding module

GBE: Glycogen branching enzyme

GDE: Glycogen debranching enzyme

GHD: Glycosyl hydrolase domain

GSD: Glycogen storage disease

LECA: Last eukaryotic common ancestor

LUCA: Last universal common ancestor

SBE: Starch branching enzyme

## Competing interests

The authors declare that they have no competing interests.

## Authors’ contributions

CZ participated in the conception and design of the study, performed the data collection, sequence analyses, phylogenetic calculations, and contributed to the interpretation of the results. AG participated in the conception and design of the study, performed the structural analyses, and contributed to the interpretation of the results. Both authors contributed to the writing of the manuscript and read and approved the final text.

## Additional files

## Supplementary Material

Additional file 1: Complete genomes analyzed. Click here for file

Additional file 2: **CBM_48- and Alpha-amylase-containing branching and debranching enzymes.** Protein identifiers (mostly from UniProt; for others, see legend for Additional file 4) for branching and debranching enzymes with CBM_48 and Alpha-amylase domains are listed (per-domain E-value cutoff: 10^-3^). For eukaryotic enzymes, taxonomic groups (such as Alveolata) are indicated. Simplified domain architecture overviews are given for each enzyme (“~” is used to indicate linkers between domains shorter than 11aa, whereas “----“stands for linkers longer than 10aa). Individual E-values for CBM_48 and Alpha-amylase domains are shown as well. Click here for file

Additional file 3: **hGDE-amylase- and GDE_C-containing eukaryotic debranching enzymes.** Protein identifiers (mostly from UniProt; for others, see legend for Additional file 4) for debranching enzymes with hGDE-amylase and GDE_C domains are listed (per-domain E-value cutoff: 10^-3^). Taxonomic groups (such as Alveolata) are indicated. Simplified domain architecture overviews are given for each enzyme (“~” is used to indicate linkers between domains shorter than 11aa, whereas “----“ stands for linkers longer than 10aa). Individual E-values for hGDE-amylase and GDE_C domains are shown as well. Click here for file

Additional file 4: **Representative examples of branching and debranching enzymes from completely sequenced genomes.** Protein identifiers are generally from the UniProt database, except for those marked with an asterisk, which are from GenBank, and those from *Sphaeroforma arctica*, *Thecamonas trahens, and Fonticula alba* which originate from the Origins of Multicellularity Sequencing Project (Broad Institute of Harvard and MIT: http://www.broadinstitute.org), and those from *Cyanidioschyzon merolae* which are from the National Institute of Genetics, Japan [69]. All examples are from completely sequenced genomes (see Additional file 1). Click here for file
